# Features of electrocerebellogram in patients with mild cognitive impairment due to Alzheimer's disease

**DOI:** 10.1002/alz70856_101432

**Published:** 2025-12-25

**Authors:** Chi Zhang, Jingping Shi, Minjie Tian

**Affiliations:** ^1^ Brain Hospital of Nanjing Medical University, Nanjing, Jiangsu, China

## Abstract

**Background:**

The cerebellum has been increasingly recognized for its critical role in the evolution of behavior and cognition in recent years. As a non‐classical cognitive brain region, the cerebellum participates in the regulation of advanced cognitive functions, such as memory, learning, executive function, and emotion, through the cerebro‐cerebellar circuits. However, research on the early diagnosis of Alzheimer's disease has predominantly focused on the cerebrum. Compared to the cerebrum, the role of the cerebellum in the onset and progression of Alzheimer's disease has been significantly overlooked. Aim of this study was to investigate the electrocerebellogram (ECeG) characteristics in patients with Alzheimer's disease across different stages and to identify sensitive biomarkers for early‐stage detection.

**Method:**

We recruited 31 healthy volunteers(HC), 40 patients with mild cognitive impairment (MCI) and 16 patients with Alzheimer's disease (AD) for this study, with continuous resting‐state EEG and ECeG recording using 71 channels to monitor cerebrum and cerebellum electrical signals. We analyzed the spectral changes in resting‐state ECeG across the three groups and compared the functional connectivity differences between the cerebellum and cerebrum in the HC and MCI groups.

**Result:**

The relative power spectrum of the cerebellum indicates that MCI group showed significantly reduced δ band power compared to both the HC and AD groups. In the θ frequency band, the MCI and AD group demonstrated higher power compared to the HC group. In the α frequency band, the power in the AD group was lower than MCI group. Functional connectivity analysis revealed stronger connectivity between the cerebellum and the frontal and parieto‐occipital lobes in the MCI group compared to the HC group.

**Conclusion:**

Alterations in the relative power of the δ and θ frequency bands of ECeG power spectrum and changes in functional connectivity between the cerebellum and cerebrum hold promise as sensitive biomarkers for the detection of MCI.

